# Letter to the Editor: Lack of requirements for conducting meta-analysis to evaluate the association between the TLR2 Arg753Gln polymorphism and the risk of sepsis

**DOI:** 10.1186/s13054-016-1410-6

**Published:** 2016-08-30

**Authors:** Yasushi Tsujimoto, Yusuke Tsutsumi, Tatsuyoshi Ikenoue

**Affiliations:** Department of Healthcare Epidemiology, Graduate School of Medicine and Public Health, Kyoto University, Yoshida Konoe-cho, Sakyo-ku, Kyoto 606-8501 Japan

We read the article published recently in *Critical Care* by Gao et al. [1] with great interest and appreciate their effort to evaluate the association between the TLR2 Arg753Gln polymorphism and the risk of sepsis. However, we would like to point out a lack of requirements for conducting meta-analysis in this study; quality assessment, appropriate search method and protocol registration.

First, the authors did not assess the quality of studies included in their meta-analysis. In general, meta-analysis of studies that are at risk of bias may be seriously misleading. If bias is present in each (or some) of the individual studies, meta-analysis will simply compound the bias of individual studies, and produce a ‘wrong’ result that may be interpreted as having more credibility [2]. Even though some of the included studies have limited information, the authors should perform sensitivity analysis excluding studies with a high risk of bias in order to explore the influence of biased studies.

Secondly, the authors explained that all available data related to potential links between the TLR2 Arg753Gln polymorphism and sepsis risk were pooled in this study. However, the authors only used free-text terms in their search strategy. To search all existent studies, it is generally recommended to use a combination of subject terms selected from controlled vocabulary and free-text terms. Using only free-text terms might reduce the search quality. In addition, the authors did not identify unpublished and ongoing studies. International trial registers should be searched to detect publication bias [2].

Lastly, the study was performed partially in accordance with the Preferred Reporting Items for Systematic reviews and Meta-Analysis (PRISMA), the protocol for which is not registered in the International Prospective Register of Systematic Reviews (PROSPERO). Registration of protocol details is now recognized as desirable in order to promote and maintain transparency in the process and to assist in minimizing the risk of selective outcome reporting bias [3].

Therefore, we conclude that Gao et al.’s meta-analysis has the potential for producing incorrect results due to a lack of quality assessment, an inappropriate search to identify relevant studies and an absence of protocol registration.

## Abbreviations

PRISMA, Preferred Reporting Items for Systematic reviews and Meta-Analysis; PROSPERO, International Prospective Register of Systematic Reviews; TLR, toll-like receptor
